# Neural Correlates of Drug-Related Attentional Bias in Heroin Dependence

**DOI:** 10.3389/fnhum.2017.00646

**Published:** 2018-01-23

**Authors:** Qinglin Zhao, Hongqian Li, Bin Hu, Yonghui Li, Céline R. Gillebert, Dante Mantini, Quanying Liu

**Affiliations:** ^1^Ubiquitous Awareness and Intelligent Solutions Lab, Lanzhou University, Lanzhou, China; ^2^Key Laboratory of Mental Health, Institute of Psychology, Chinese Academy of Sciences, Beijing, China; ^3^Department of Experimental Psychology, University of Oxford, Oxford, United Kingdom; ^4^Department of Brain and Cognition, KU Leuven, Leuven, Belgium; ^5^Research Centre for Motor Control and Neuroplasticity, KU Leuven, Leuven, Belgium; ^6^Neural Control of Movement Lab, ETH Zurich, Zurich, Switzerland

**Keywords:** dot-probe task, event-related potentials, attentional bias, source localization, heroin-related cues, P3

## Abstract

The attention of drug-dependent persons tends to be captured by stimuli associated with drug consumption. This involuntary cognitive process is considered as attentional bias (AB). AB has been hypothesized to have causal effects on drug abuse and drug relapse, but its underlying neural mechanisms are still unclear. This study investigated the neural basis of AB in abstinent heroin addicts (AHAs), combining event-related potential (ERP) analysis and source localization techniques. Electroencephalography data were collected in 21 abstinent heroin addicts and 24 age- and gender-matched healthy controls (HCs) during a dot-probe task. In the task, a pair of drug-related image and neutral image was presented randomly in left and right side of the cross fixation, followed by a dot probe replacing one of the images. Behaviorally, AHAs had shorter reaction times (RTs) for the congruent condition compared to the incongruent condition, whereas this was not the case in the HCs. This finding demonstrated the presence of AB towards drug cues in AHAs. Furthermore, the image-evoked ERPs in AHAs had significant shorter P1 latency compared to HCs, as well as larger N1, N2, and P2 amplitude, suggesting that drug-related stimuli might capture attention early and overall require more attentional resources in AHAs. The target-related P3 had significantly shorter latency and lower amplitude in the congruent than incongruent condition in AHAs compared to HCs. Moreover, source localization of ERP components revealed increased activity for AHAs as compared to HCs in the dorsal posterior cingulate cortex (dPCC), superior parietal lobule and inferior frontal gyrus (IFG) for image-elicited responses, and decreased activity in the occipital and the medial parietal lobes for target-elicited responses. Overall, the results of our study confirmed that AHAs may exhibit AB in drug-related contexts, and suggested that the bias might be related to an abnormal neural activity, both in early and late attention processing stages.

## Introduction

Drug-related attentional bias (AB), the effect for which substance-addicted patients involuntarily orient their attention toward drug-related cues, has been considered a fundamental factor in substance abuse, addiction development and maintenance (Mckay, 1999; Franken et al., 2003; Field and Cox, 2008; Robinson and Berridge, 2008). AB has been observed in various types of addictions, including alcohol (Townshend and Duka, 2001), cigarette (Chanon et al., 2010), heroin (Waters et al., 2012), and cocaine (Mayer et al., 2016), even in abstinent individuals (Noël et al., 2006; Rahmanian et al., 2006; Wang et al., 2007). For example, previous studies have shown that abstinent heroin addicts (AHAs) exhibit AB to heroin-related stimuli (Marissen et al., 2006). However, few studies investigated the underlying neural correlates of AB for heroin-related stimuli in AHAs. The dot-probe task, developed by MacLeod et al. (1986), is a widely used paradigm to investigate AB (Norman et al., 2014; Ursache and Blair, 2015). It is based on the observation that subjects tend to respond faster to a probe stimulus that is presented in an attended rather than unattended area (Franken et al., 2000). Recently, the task has been extended to investigate AB in cigarette (Ehrman et al., 2002; Spencer, 2015), alcohol (Klein et al., 2013; Mcateer et al., 2015; Clerkin et al., 2016), as well as drug dependence (Lubman et al., 2000; Bradley et al., 2003; Field et al., 2009; Gardini, 2009).

Electroencephalography directly measures neural activity, which can be used to investigate information processing and functional interactions in the human brain with millisecond resolution (Liu et al., 2017). Particularly, the high temporal resolution of event-related potentials (ERPs) allows us to examine sequential cognitive processing states involved in a task. For example, early visual components approximately 80–250 ms after the stimuli onset, P1 or N1, are typically associated with the lower-order visual processing (Omoto et al., 2010), such as the identification of stimuli and their global encoding process (Warbrick et al., 2014), whereas late components from 250 to 500 ms, P2, N2, and P3, are thought to reflect higher-order cognitive processes (Michalewski et al., 1986; Kanske et al., 2011; Ibanez et al., 2012; Kompatsiari et al., 2016), such as selective attention processing, conflict processing, stimulus categorizing, and inhibition processes (Luck et al., 1990; Bocquillon et al., 2014). The P3 component is a positive deflection with a peak around 300 ms after stimulus onset (Herrmann and Knight, 2001), which is related to selective attention processes. Overall, ERPs permit to explore the neural basis of cognitive processes with high sensitivity and reliability, and are complementary to behavioral analyses conducted, for instance, by measuring reaction times (RTs) (Kappenman et al., 2014).

Previous brain imaging studies, using positron-emission tomography (PET) and functional magnetic resonance imaging (fMRI) techniques, have reported that the brain regions that are most vulnerable to heroin addiction are specific prefrontal, parietal, occipital, and temporal regions and subcortical regions (Kilts et al., 2001) linked with reward, motivation/drive, memory/learning, inhibition as well as emotional control (Pandria et al., 2016). AB to drug-related cues generally activates parts of the prefrontal cortex that are relevant to attentional processing (Goldstein and Volkow, 2011), such as dorsolateral prefrontal cortex (dlPFC), the anterior cingulate cortex (ACC), and the inferior frontal gyrus (IFG). However, the low temporal resolution of PET and fMRI does not allow us to disentangle fast cognitive processes underlying AB. In this regard, the EEG source localization technique can be utilized to explore the underlying neural changes of drug-related AB and the associated brain regions (Field and Cox, 2008; Janes et al., 2010; Crunelle et al., 2012).

The aim of this study is to investigate the neural abnormalities of drug-related AB in heroin dependence using a dot-probe task, combining ERP analyses, and source localizations. We hypothesize that AHAs would respond faster than healthy controls (HCs) to the dots that replace drug-related stimuli compared to neutral stimuli. Furthermore, we expect that source analysis of ERP components in AHAs would provide electrophysiological evidences for abnormalities in cognitive processing related to AB.

## Materials and Methods

### Participants

We enrolled 45 participants (all males) in the study, including 21 AHAs and 24 HCs. The AHAs (age: *M* = 37.33 years, *SD* = 7.18 years) were recruited from the Gansu Compulsory Isolated Detoxification Center in China, meeting the criteria of Diagnosis and Statistics of Mental Disorder 5th edition (DSM-V) for heroin dependence. The AHAs who participated in our study were abstinent from heroin and other dependent drugs for at least 1 month (abstinent period: *M* = 4.43 months, *SD* = 4.42 months). The HCs (age: *M* = 35.29 years, *SD* = 8.11 years) were recruited from the local community, and had no history of alcohol or drug abuse. These two groups showed no significant difference in the age [*t*(43) = 0.889, *p* = 0.379], but the educational level was significantly lower in AHAs (*M* = 2.62, *SD* = 2.75 years) compared to HCs (*M* = 6.71, *SD* = 3.7 years) [*t*(43) = -4.16, *p* < 0.05]. All the subjects were right-handed, had normal or corrected-to-normal visual acuity, and no history of neurological problems. None of the subjects were taking any psychotropic, neurological, or psychiatric medications at the time of experiment. All participants gave written informed consent before participating in the study, which had been approved by the Ethics Commission of Institute of Psychology of Chinese Academy of Sciences (Approval Number: H15020).

### Stimuli

We selected drug-related images and neutral scenic images as stimuli to be used in the dot-probe task. We initially selected 60 images from Institute of Psychology of the Chinese Academy of Sciences, including 30 heroin-related images and 30 neutral images. The heroin-related stimuli were images of drug paraphernalia and scenes of an unidentified addict injecting drugs. All stimuli were matched for brightness, contrast, and color. The images were rated on a scale from one to nine by heroin addicts (*N* = 29) who met the addiction criteria of DSM-V and had no history of neurological problems. The 10 images with the highest scores (score: 7.91 ± 0.11) were selected as drug-related cues, and 10 images with the lowest scores (score: 1.42 ± 0.23) were selected as neutral images. Notably, the scores of the drug-related images were significantly higher than those of the neutral images [unpaired *t*-test, *t*(18) = 79.5, *p* < 0.005].

### Procedure

The experiment was performed in a quiet, air-conditioned laboratory with dimly natural light. The participants were seated comfortably in front of a 21-inch computer screen. To reduce excessive eye movements and blinks, participants were instructed to keep fixation on the center of screen during experiment.

**FIGURE 1:**
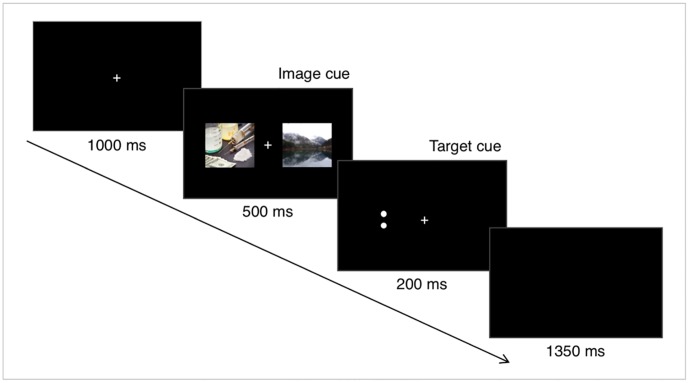
Experimental paradigm. Each trial began with a fixation cross with 1000 ms duration. Immediately following offset of the fixation cross, a drug-related image and a neutral image were presented bilaterally for 500 ms. Next, a target stimulus, which was either a horizontal or a vertical pair of dots, was shown for 200 ms. The trial ended with a 1350 ms intertrial interval.

The dot-probe task was programmed and presented using E-Prime 2.0 (Psychology Software Tools, Inc.). The experimental paradigm was shown in **Figure 1**. Specifically, each trial began with a fixation cross (1 cm × 1 cm) in the center of the screen for 1000 ms. Immediately following offset of the fixation cross, a pair of images was presented for 500 ms. Each pair contained a drug-related image and a neutral image. In each pair, one of the stimuli appeared to the left of the fixation cross and one appeared to the right, with a visual angle of 10 degrees. The location of the drug-related image was randomized across trials. The images were immediately followed by the target stimulus, which was either a horizontal pair of dots or a vertical pair. Each dot had 5 mm center distance, with 1 mm radius. The target stimuli remained 200 ms. The participants were asked to judge whether the dots were oriented vertically or horizontally, and to press the response key as soon as possible. They were instructed to press the button by using the middle finger and the index finger of the right hand. If the answer was incorrect or took longer than 1000 ms, the screen showed a feedback warning (‘X’ or ‘?’), whereas no feedback was present if the response was correct and fast enough. During the intertrial interval, which lasted 1350 ms, a black screen without fixation cross was presented.

There were four kinds of target stimuli: (1) drug-related cue and target both in the left visual field, (2) drug-related cue and target both in the right visual field, (3) drug-related cue in the left, and target in the right visual field, and (4) drug-related cue in the right, and target in the left visual field. Each condition was presented 60 times, resulting in a total of 240 trials. The first two conditions, in which the drug-related cue and target are in the same side, are referred to as the congruent (CON) condition, and the other two, in which the drug-related cue and target are in the different sides, as the incongruent (INCON) condition.

Before the real experiment, the participant had one or more practice runs (20 trials each), during which EEG was not recorded, until he/she reached a response accuracy of 80%. The real experiment was composed of three runs. Each of these had 80 trials, and lasted about 6 min.

### EEG Recording and Processing Procedures

EEG signals were recorded using a 64-channel electrode cap (Brain Products, Gilching, Germany) with International 10/20 montage. The scalp impedance of each sensor was kept below 10 kΩ, as suggested by the manufacturer. The EEG signals were recorded at a sampling rate of 5000 Hz with the vertex electrode as reference, and filtered in the band 0.01–100 Hz.

Signal processing and analysis of the EEG data was performed using BrainVision Analyzer 2.0 (Brain Products, Gilching, Germany). The raw EEG signals were resampled at 1000 Hz and then band-pass filtered at 1–40 Hz with a FIR filter. Independent component analysis (ICA) was used to remove the ocular and muscle artifacts (Delorme and Makeig, 2004). The cleaned EEG signals were re-referenced using the average reference.

### ERP Calculation

EEG data were segmented into epochs from 100 ms before image onset to 500 ms after image onset. The pre-stimulus was used for baseline correction. In addition, the EEG data were segmented into epochs starting 700 ms before the dot stimulus onset, which is 200 ms before image onset, and ending 1000 ms after dot stimulus onset. In the latter case, epochs were baseline corrected in the time window from 700 to 500 ms before dot stimulus onset. Due to the carryover effects of image stimulus, the average voltage of 200–0 ms before dot stimulus onset biased, was thus not suitable for the baseline (Supplementary Figure S2). Trials with a feedback warning, which was present in the case of incorrect behavioral response, were excluded. The EEG epochs with absolute voltage value exceeding 100 μV were also excluded from analyses. The trials of single ERP waveforms superimposed were not less than 40 for each condition and per subject (57.43 ± 0.42 for image-locked P1, N1, P2, and N2; 114.38 ± 0.79 for target-locked P3).

### Analysis of ERP Components

In this study, we investigated ERP components and the corresponding source-space activity to clarify the neural correlates of drug-related AB in heroin dependence. Specifically, we examined five ERP components associated with different stages of attention processing: image-elicited P1, N1, P2, and N2, and target-elicited P3 (Carretié et al., 2004; Thai et al., 2016). Since the image-elicited response lasted relatively long, the early components for target-elicited response were severely distorted by the image-elicited response. Therefore, early ERP components elicited by target (dot) stimulus were not considered in this study.

We calculated image-elicited P1, N1, P2, and N2 in two conditions, referring to drug-related cue either on the left or on the right. P1 was defined as the first positive peak within a 20 ms time window around the P1 peak (the ‘peak window’) after picture onset for each subject. N1 was defined as the first negative peak within 50 ms around the peak identified in the time window from 170 to 220 ms. P1 and N1 were examined at the electrode O1, PO3, PO7 or O2, PO4, PO8, in the hemisphere contralateral to the drug-related cue, considering the effect of optic chiasm in the early visual response. In addition, P2 was measured by averaging activity in the time window 240–320 ms after image onset at O1, O2, Oz, PO3, PO4, PO7, and PO8 electrodes. N2 was defined as the second negative peak in the time window 250–350 ms after the image onset at FC1, FC2, FC3, FC4, FCz, C1, C2, C3, C4, and Cz electrodes. We also examined the target-related P3, the most prominent ERP component related to attentional processes (Verleger, 1988). P3 was defined by the average activity in a 100 ms time window between 300 and 400 ms at CP1, CP2, CP3, CP4, CPz, P1, P2, P3, P4, and Pz electrodes, for congruent and incongruent conditions.

### ERP Source Localization

To identify the brain regions involved in AB and their specific role in attentional processing, we reconstructed the ERP sources (Pascualmarqui et al., 2011) and compared neuronal activity between two groups in the same condition (between-subject comparison) or between different conditions in the same group (within-subject comparison). A forward head model was built by using the boundary element method (BEM), using a MNI152 template (Fuchs et al., 2002; Pascual-Marqui, 2002) and standard electrode positions. Then, the activity at each brain voxel was estimated by exact low-resolution brain electromagnetic tomography (eLORETA) using the sLORETA and eLORETA software package (Pascual-Marqui, 2002; Pascualmarqui et al., 2011). eLORETA has been demonstrated to have lower localization error compared to LORETA (Jatoi et al., 2014) and to be suitable for accurate EEG source localizations (Zhao et al., 2017). The brain sources were constrained to be in the cortical gray matter, resulting in 6239 voxels at 5 mm resolution.

To enhance the spatial sensitivity of the ERP procedure, we used the following time windows on the EEG source analysis: P1 (the 20-ms peak window), N1 (170–220 ms), P2 (240–320 ms), N2 (250–350 ms), and P3 (the 100-ms peak window). Source reconstruction was performed for each experimental condition (image stimulus and target stimulus) and group (AHA and HC), respectively. To be noted, the sources were computed in the frequency range 1–40 Hz. It is important to note that, given the ill-posedness of EEG source localizations, the maps presented in this study should be considered rough estimates of the brain sources during the dot-probe task.

### Statistical Analysis

For the behavioral results, a 2 × 2 Analysis of variance (ANOVA) was performed on the RTs for correct responses, with the group (AHA vs. HC) as between-subjects factor and target-stimuli condition (congruent vs. incongruent) as within-subjects factor.

Statistical analyses of ERP components were performed with SPSS 19.0 (IBM, Armonk, NY, United States). We used an ANOVA to investigate if there were differences between AHA and HC. We performed a test of homogeneity of variances, and adjusted *F* values using Brown–Forsythe’s and Welch’s corrections if necessary. For repeated-measure ANOVA, the Mauchly’s test was used to test for sphericity, and the Greenhouse-Geisser correction was applied if necessary.

For the image-elicited P1, N1, P2, and N2 components, we performed a 2 × 2 repeated-measure ANOVA with group (AHA vs. HC) as a between-subjects factor and position of the drug-related cues (left vs. right) as within-subjects factors. For the target-elicited P3 component, repeated measures ANOVA were performed with target-stimuli (congruent vs. incongruent) as within-subjects factor and group (AHA vs. HC) as between-subjects factor. The statistical significance was set to *p* = 0.05 with family-wise error (FWE) correction for multiple comparisons.

Group-level source images were generated by using group as between-subject factor in each condition. ANOVA was calculated to examine significance differences per time period and per condition within each group (AHA or HC) and between groups (AHA vs. HC). The statistical significance level was set to *p* = 0.05. In addition, voxel-wise *t*-tests (two-tailed) were performed to compare current density between conditions in each group and between groups.

## Results

**FIGURE 2:**
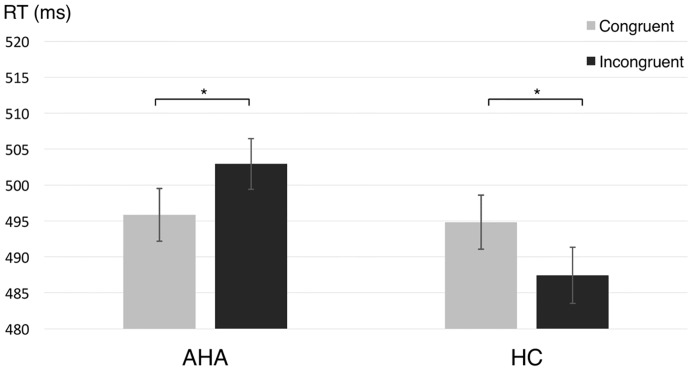
Reaction time (RT) for abstinent heroin addict (AHA) and healthy control (HC) groups in congruent and incongruent conditions, respectively. The reaction times (RTs) in the congruent condition, as compared to the incongruent condition, was significantly shorter for AHAs (*p* = 0.044) and significantly longer for HCs (*p* = 0.040). Error bars denote standard error. ^∗^*p* < 0.05.

The task performance, measured by accuracy rate, for AHAs (92.64 ± 4.61%) and HCs (92.82 ± 4.02%) showed no significant difference [*t*(43) = 0.0283, *p* = 0.9776], implying that the difference in the educational level between AHAs and HCs did not affect task performance. A 2 × 2 ANOVA on the RTs showed no significant main effect for group or condition (congruent, incongruent), respectively [group: *F*(1,43) = 0.107, *p* = 0.745; condition: *F*(1,43) = 0.004, *p* = 0.95], whereas the group × condition interaction was significant [*F*(1,43) = 8.03, *p* = 0.007]. Moreover, RTs in different conditions were significantly different both for AHAs [*F*(1,43) = 3.61, *p* = 0.044] and for HCs [*F*(1,43) = 4.448, *p* = 0.04]. Specifically, AHAs tended to have quicker response to targets preceded by drug-related cues compared to targets preceded by neutral images, whereas the opposite pattern was observed in HCs (**Figure 2**).

**FIGURE 3:**
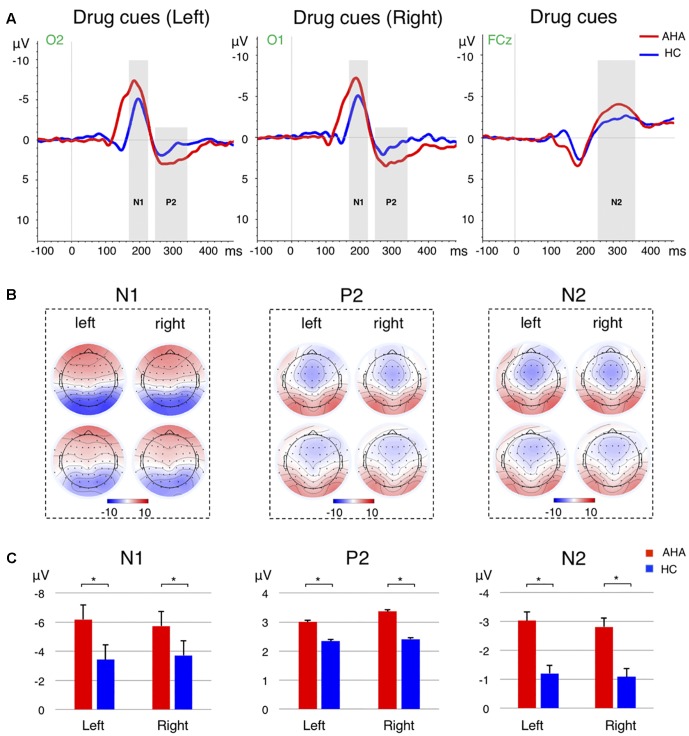
Event-related potential (ERP) analysis for the image-related responses. **(A)** The grand average ERP waveforms for the image-related responses from the selected electrode for the drug cues in the left (Left), drug cues in the right (Middle), and the averaged across left and right cases (Right). The N1 and P2 ERPs for the drug cues presented in the left are from the O2 electrode, whereas the ERPs for the drug clues in the right are from the O1 electrode. The N2 waveforms are extracted from the FCz electrode. The time windows for N1, P2, and N2 are marked by the gray shadow, where were 170–220 ms, 240–320 ms, and 250–350 ms, respectively. The red and blue lines refer to AHA and HC group, respectively. **(B)** Scalp topography of the N1 (Left), P2 (Middle), and N2 (Right) components for left and right drug cues, for AHA and HC groups, respectively. The components are averaged across subjects and time windows. **(C)** Bar plots show mean and standard error of the intensity of N1 (Left), P2 (Middle), and N2 (Right) components for AHA group (red) and HC group (blue), respectively. Error bars denote standard error. ^∗^*p* < 0.05.

**FIGURE 4:**
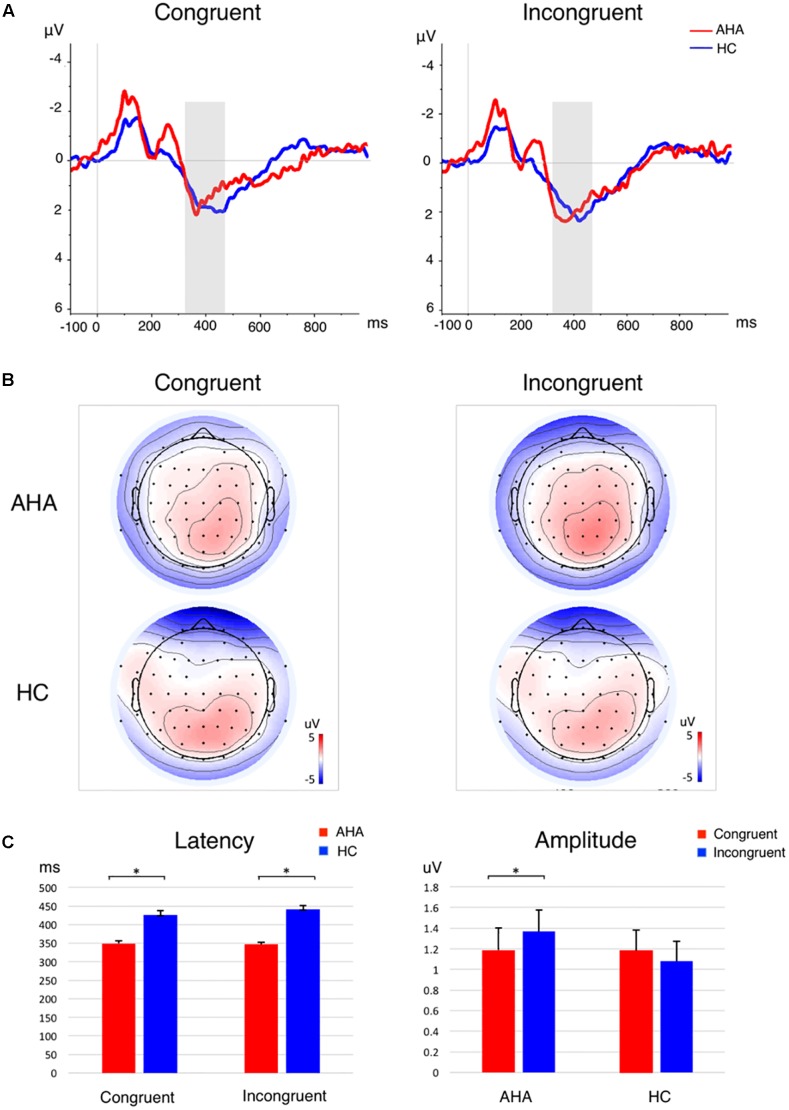
P3 component for the target-related responses. **(A)** The grand average ERP waveform in congruent (Left) and incongruent condition (Right). The P3 time window is marked by a gray shadow. The AHA group is depicted in red, and the HC group in blue. **(B)** Scalp topography of P3 component per group per condition. The P3 component is averaged across subjects and time windows. **(C)** Bar plots for P3 latency (Left) and amplitude (Right) per group per condition. Error bars denote standard error. ^∗^*p* < 0.05.

**FIGURE 5:**
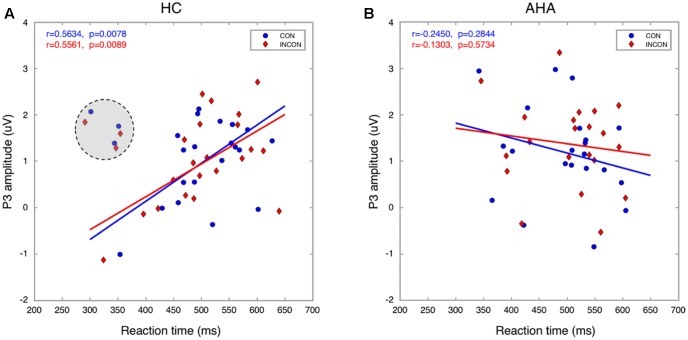
Relationship between P3 amplitude and RT. The P3 amplitude is significantly correlated with RT in both congruent and incongruent conditions for HCs **(A)**. In contrast, it was anticorrelated with RT for AHAs **(B)**. Three HCs (marked with a gray shadowed circle) were excluded from the analysis as they were considered outliers.

To investigate the effects of drug-related cues on the allocation of attentional resources, we compared the ERP components between AHAs and HCs. In particular, we investigated the image-elicited P1 (Supplementary Figure S1), N1, P2, and N2 (**Figure 3**), and target-elicited P3 (**Figure 4** and Supplementary Figure S2). Using a repeated-measure ANOVA, we observed a main effect of group on P1 latency [*F*(1,43) = 15.246, *p* < 0.001], N1 amplitude [*F*(1,43) = 4.418, *p* = 0.041], P2 amplitude [*F*(1,43) = 5.336, *p* = 0.026], N2 amplitude [*F*(1,43) = 19.486, *p* < 0.001], and P3 latency [*F*(1,43) = 25.683, *p* < 0.001], but not on P1 amplitude [*F*(1,43) = 0.423, *p* = 0.519] or P3 amplitude [*F*(1,43) = 0.676, *p* = 0.416]. Condition (congruent vs. incongruent) and group (AHA vs. HC) had a significant interaction effect on P3 amplitude [*F*(1,43) = 7.140, *p* = 0.011], but not on P3 latency [*F*(1,43) = 0.489, *p* = 0.488]. For P3 amplitude, the congruent or incongruent condition showed a significant effect on P3 amplitude for the AHA group [*F*(1,43) = 8.08, *p* = 0.007], but not the HC group [*F*(1,43) = 0.76, *p* = 0.388] (**Figure 4C**). Importantly, the amplitudes of target-elicited P3 showed significantly positive correlation with RTs in both congruent (*r* = 0.5634, *p* < 0.01) and incongruent conditions (*r* = 0.5561, *p* < 0.01) for HCs (**Figure 5A**). Surprisingly, anti-correlations were obtained in both congruent condition (*r* = -0.2450, *p* = 0.2844) and incongruent condition (*r* = -0.1303, *p* = 0.5734) for AHAs, although they did not reach significance (**Figure 5B**). Notably, we did not find any significant correlation between RT and withdrawal time (*r* = -0.063, *p* = 0.787 for congruent condition; *r* = 0.117, *p* = 0.612 for incongruent condition), neither between P3 amplitude and withdrawal time (*r* = -0.040, *p* = 0.865 for congruent condition; *r* = -0.132, *p* = 0.570 incongruent condition).

**FIGURE 6:**
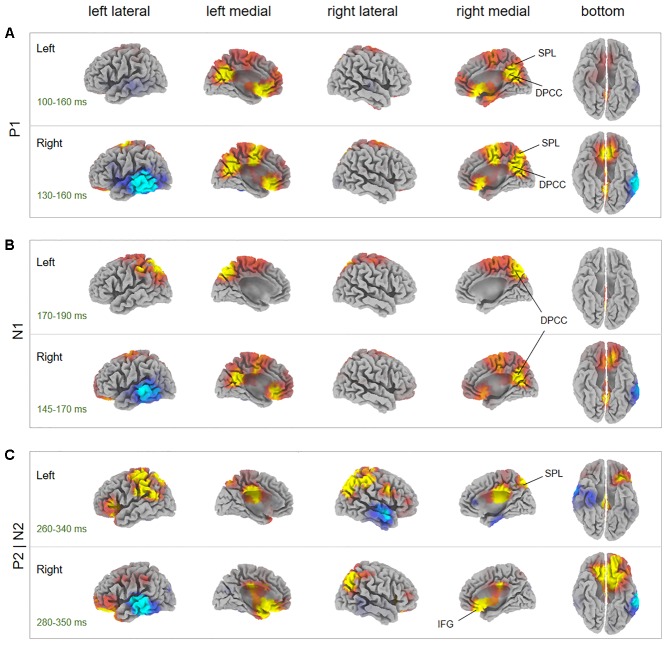
Between-subject comparisons for image-elicited ERP sources. The *t*-score maps for the comparison between AHAs and HCs for P1 component **(A)**, N1 component **(B)**, and P2 and N2 component **(C)** are shown for drug-related cues presented in the left and right visual fields, respectively. The brain regions with yellow/red color indicate AHAs > HCs, whereas green/blue color indicates AHAs < HCs. The time periods for different components are indicated as well. To be noticed, the time window for P2 and N2 components are overlapping. Significant brain regions (*p* < 0.05, FWE corrected) were indicated on the map. The peak MNI coordinate regions for each comparison are reported in **Table 1**. dPCC, dorsal posterior cingulate cortex; IFG, inferior frontal gyrus; SPL, superior parietal lobule.

**FIGURE 7:**
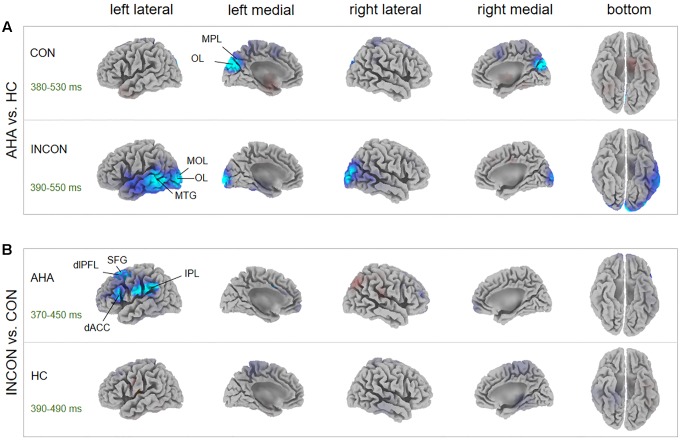
P3 Source analysis. **(A)** The *t*-score maps for the comparison between AHAs and HCs are shown in the congruent (Up) and incongruent condition (Bottom), respectively. The brain regions with green/blue color indicate AHAs < HCs. The significant differences between brain regions [*p* < 0.05, family-wise error (FWE) corrected] were indicated in the map. The peak MNI coordinates for the contrast between groups are reported in **Table 1**. **(B)** *T*-score maps for the comparison between congruent and incongruent conditions for AHA group (Up) and HC group (Bottom), respectively. The brain regions with red color indicate CON > INCON, whereas those with blue color indicate CON < INCON. The peak MNI coordinates for the contrast between conditions are reported in Supplementary Table S1. dACC, dorsal anterior cingulate cortex; dlPFL, dorsal-lateral prefrontal lobe; IPL, inferior parietal lobe; MOL, medial occipital lobe; MPL, medial parietal lobe; MTG, middle temporal gyrus; OL, occipital lobe; SFG, superior frontal gyrus; CON, congruent; INCON, incongruent.

Source localization revealed several brain regions for image-related ERP components (i.e., P1, N1, P2, N2) of interest (**Figure 6**, **Table 1**, and Supplementary Table S1). During the P1 time window, dorsal posterior cingulate cortex (dPCC) and superior parietal lobe (SPL) were significantly more active in AHAs than in HCs, whether the drug-related cue was presented in the left or right hemi-spatial field. Also, strong neuronal activity in dPCC in AHAs was maintained until the N1 response. For the P2 and N2 time windows, we found significant clusters of differential activation in the SPL and IFG for drug-related cue both in the left or right hemi-spatial fields. We then examined the neural sources associated with target-related P3 activity (**Figure 7**, **Table 1**, and Supplementary Table S1). We observed the medial parietal lobe and occipital lobe in AHAs to be significantly less active both in congruent and incongruent conditions, whereas brain activity in MTG was reduced for AHAs in the incongruent condition, but not the congruent condition. A within-subject comparison showed reduced activity in superior frontal gyrus (SFG), dorsolateral prefrontal lobe, dorsal anterior cingulate cortex (dACC) and IPL for AHAs in the incongruent compared to the congruent condition, whereas no brain regions showed differential activity between two conditions for HCs.

**Table 1:** Peak MNI coordinates for between-subject factor comparison of between abstinent heroin addicts (AHAs) and healthy controls (HCs).

Component	Time (ms)	Condition	Coordinate region	Peak MNI coordinate			*P*-value
P1	90–160	LEFT	dPCC	0	-57	26	0.0256^∗^
			SPL	0	-57	30	
		RIGHT	dPCC	5	-52	35	0.0172^∗^
			SPL	0	-57	30	
N1	150–220	LEFT	dPCC	10	-52	30	0.0446^∗^
		RIGHT	dPCC	-15	-57	30	0.0386^∗^
P2| N2	260–350	LEFT	SPL	40	-61	49	0.0396^∗^
		RIGHT	IPFG	-30	19	-5	0.0438^∗^
P3	380–550	CON	OL	-5	-80	25	0.0132^∗^
			MPL	-5	-75	25	
		INCON	MOL	0	-100	-5	0.0264^∗^
			OL	-5	-100	0	
			MTG	-65	-50	0	

*Clusters surviving threshold of p < 0.05 family-wise error (FWE)-corrected. Abbreviations: dPCC, dorsal posterior cingulate cortex; IPFG, inferior prefrontal gyrus; MPL: medial parietal lobe; MOL: medial occipital lobe; OL: occipital lobe; MTG: middle temporal gyrus; SPL, superior parietal lobule; CON: congruent; INCON: incongruent. ^∗^p < 0.05.*

## Discussion

The present study explored the neural correlates of drug-related AB in AHAs by examining ERP components in a dot-probe task. Our behavioral results confirmed the hypothesis that AHAs respond faster than HCs to dots that replace drug-related stimuli, as compared to neutral stimuli. More importantly, the influence of the drug-related cue on attentional processing was reflected by altered neural responses in the early (sensory) stage, as indexed by the P1, N1, P2 responses, but also the late (cognitive) processing stage, as indexed by the N2 and P3 responses. These findings provided novel insights into the neural mechanisms underlying AB toward the drug-related cues in AHAs.

In line with a previous study (Constantinou et al., 2010), we found behavioral evidence of an AB to drug in the AHA group using the traditional RT measure of drug-related bias in a dot-probe task (i.e., the difference in RT on congruent and incongruent trials). Specifically, we observed faster RTs in congruent as compared to incongruent conditions in AHAs, suggesting that the attention of heroin addicts was attracted by drug-related images even after a certain withdrawal period (**Figure 2**). Previous studies showed that drug-related cues could produce a strong subjective craving in AHAs, which may result in AB (Franken, 2003; Lubman et al., 2008). On the contrary, HCs showed longer RTs in the congruent compared to the incongruent condition, which might be due to an intrinsic avoidance response to drugs (Banerjee, 1971).

The ERP analysis permitted to identify distinct neural responses associated with drug-related AB in AHAs. Visual information processing may be characterized by four different ERP components, related to sensory encoding (around about 80 ms), early categorization (around about 100 ms), and stimulus recognition (around about 150 ms) (Richards, 2003; Lithfous et al., 2014; Oren et al., 2016), and spatial orienting and visual short-term memory (VSTM) (Nobre et al., 2008; Kuo et al., 2014) (around about 250 ms). We observed a significantly smaller P1 latency in AHAs than HCs, suggesting that the manifestation of AB started from an early stage of stimulus categorization and drug contexts might be encoded more quickly in AHAs (Supplementary Figure S1A). However, we did not find significant differences in the contralateral P1 amplitude between AHAs and HCs. This result might be explained by the presence of drug-related image and neutral scenery image bilaterally. Moreover, the source localization for P1 mainly identified dPCC and SPL (**Figure 6**). This is in good agreement with other studies showing that dPCC is associated with stimulus encoding (Tucker et al., 2011), and SPL is involved in maintaining a spatial reference system for goal oriented behavior. It may be associated with spatial integration of visual features (Wilkinson et al., 2002; Cornette et al., 2006; Molenberghs et al., 2016) and be also related to attentional shifting (Molenberghs et al., 2007; Vandenberghe et al., 2012). More generally, it is involved in the compilation of an attentional priority map (Gillebert et al., 2012). Unlike the P1 amplitude reflecting the inhibition, the N1 amplitude reflects the amount of initial input to attentional resources to the cues (Li et al., 2014). Our study showed significantly larger N1 amplitude in AHAs than in HCs, especially at parietal and occipital electrodes (**Figures 3A,B**). Visual spatial attention signals from parietal to occipital cortex enable top-down attention processing (Lauritzen et al., 2009). Accordingly, more attention may be allocated to drug-related cues in AHAs in bottom-up attention process. In line with previous studies (Rosazza et al., 2009), we found the N1 topography to be characterized by a typical bilateral posterior negativity, which is consistent with sources located in bilateral occipital–temporal regions (**Figure 3B**, left panel). More specifically, we found dPCC to be hyperactive in AHAs in the N1 time window (**Figure 6B**). dPCC is functionally connected with dorsal attention regions (Campbell et al., 2013) and involved in memory loading for the stimulus processing (Oren et al., 2016) and selective processing of external task-relevant information (Campbell et al., 2013).

The parieto–occipital P2, which was evoked at the latency of around 280 ms by image cues, has been considered to be related to memory performance (Dunn et al., 1998) and working memory (Lefebvre et al., 2005). In turn, N2 is typically associated with response to previous memorized stimuli (Hu et al., 2013) and cognitive control of response inhibition. Its neural sources are most likely located in dACC (Nieuwenhuis et al., 2003; Botvinick et al., 2004). In line with previous studies (Howard and Chaiwutikornwanich, 2006; Pinal et al., 2014; Gajewski and Falkenstein, 2015), we observed larger P2 amplitude and N2 amplitude in AHAs, possibly indicating the reinforcement of the memory related to drugs and an increased allocation of attention to drug-related stimulus in AHAs. Source localization for P2 and N2 period mainly identified SPL and IFG (**Figure 6C**). IFG, a region involved in ventral attention network, is thought to play an important role in stimulus-driven orientation of covert visual spatial attention (Corbetta et al., 2000; Serences et al., 2005). Concurring with previous studies (Luedke et al., 2013), stronger activity in SPL and IFG areas of AHAs during N2 and P2 periods might relate to filtering of irrelevant stimuli.

The analysis of ERPs following the presentation of dot stimuli permitted to investigate how drug-related attention bias affected cognitive processing (Field et al., 2009; Lobben and D’Ascenzo, 2015). Previous studies considered the P3 as an endogenous psychological component, a sign of processes of memory access evoked by evaluation of stimuli in tasks requiring a covert or an overt response (Donchin, 1979, 1981; Polich, 2007). In particular, the latency of P3 is associated with the evaluation of stimuli and strategy adjustment for subsequent processing steps (Donchin, 1979, 1981). Our results showed shorter P3 latency in AHA compared to HC (**Figures 4A,C** left panel), reflecting a shorter time required for the evaluation or classification of the stimulus in AHAs, and lower P3 amplitude in AHAs in the congruent compared to the incongruent (**Figures 4C** right panel, **5B**). This suggests that working memory in AHAs is slowly updated in the congruent condition (Donchin, 1979, 1981). P3 amplitudes were found to be positively correlated with RTs (**Figure 5A**) for HCs, which is well in line with the concept that larger P3 amplitude reflects greater perceptual load (Wu et al., 2009; Wang et al., 2014) and higher distribution of psychological resources (Polich, 2007). This positive correlation was lost in AHAs (**Figure 5B**). This might be caused by altered attention processes in AHAs (Wang et al., 2014). Previous EEG studies showed that the neurophysiological activity of P3 wave emerge from bilateral occipital areas during visual attention and visual memory cognitive tasks (Coullaut-Valera García et al., 2007), and fMRI studies indicated that the medial wall of the SPL may contribute to bottom-up visual integration (Pflugshaupt et al., 2016). Concurring with previous studies, our P3 results suggested that the drug-related AB in AHAs might largely affect bottom-up attention processes and memory cognitive responses relevant to the dot stimulus. Importantly, the effects of withdrawal treatment on the bottom-up attention processes (indexed by P3 amplitude or by RT) might not be simply dependent on the withdrawal time, since neither RT nor P3 amplitude was correlated with withdrawal time. Notably, significant differences in the SFG, dorsal-lateral prefrontal cortex (dlFPC), dACC, and IPL were found between the congruent and incongruent conditions in AHAs, but not in HCs (**Figure 7**). This indicates differences in AHAs between congruent and incongruent conditions. Previous fMRI studies showed that the response inhibition process in heroin addiction is associated with abnormal brain activity in dACC and SFG (Lee et al., 2005; Fu et al., 2008; Schmidt et al., 2014). dlPFC is known to be involved in the generation of the P3 component, which reflects top-down processes as stimulus categorization and voluntary decision-making (Bocquillon et al., 2014; Kuo et al., 2014). These results may help explaining why AHAs easily relapse again.

Although our study led to a series of findings that might contribute to a better understanding of attention bias in heroin dependence, some limitations need to be noted. First, the low educational level of subjects, in particular for AHAs, might have an impact on their performance in the task. Second, for the experiment protocol, we did not use a varying time for image presentation, neither a jittered intertrial interval. This may raise questions regarding the extent to which neural and behavioral effects that are attributed to attention are confounded by perceptual expectations. Third, we did not track eye-movements during the experiment. Eye-tracking technology, a non-invasive method for measuring gaze, has been proven to be a useful tool to investigate the visual AB (Shechner et al., 2013; Garcíablanco et al., 2014; Fashler and Joel, 2016), which can be considered in future studies. Fourth, the low-density EEG montage and the use of a volume conduction template might have limited the spatial resolution and the accuracy of source reconstruction (Liu et al., 2017). Finally, we found that early visual stimulus processing affects subsequent cognitive processing, as measured by RT. Future studies are therefore warranted to disentangle effects of attention and prediction on early stimulus processing.

## Conclusion

In this study, we investigated the neural correlates of drug-related attention bias in AHAs. We revealed that primary differences compared to healthy individuals are already coded in early stimulus encoding and recognition. Moreover, late responses were also aberrant, and possibly related to impaired stimulus evaluation and inhibition. Together, these findings may contribute to a better understanding of the neural basis of attention bias.

## Author Contributions

BH and QL: study conception and design. QZ, HL, and YL: acquisition of data. HL, BH, CG, DM, and QL: analysis and interpretation of data. All authors have drafted the manuscript. CG, DM, and QL: critical revision.

## Conflict of Interest Statement

The authors declare that the research was conducted in the absence of any commercial or financial relationships that could be construed as a potential conflict of interest. The reviewer LK and handling Editor declared their shared affiliation.

## References

[B1] Banerjee U. (1971). Acquisition of conditioned avoidance response in rats under the influence of addicting drugs. *Psychopharmacology* 22 133–143. 10.1007/BF00403621 5166348

[B2] Bocquillon P. Bourriez J. L. Palmero-Soler E. Molaee-Ardekani B. Derambure P. Dujardin K. (2014). The spatiotemporal dynamics of early attention processes: a high-resolution electroencephalographic study of N2 subcomponent sources. *Neuroscience* 271 9–22. 10.1016/j.neuroscience.2014.04.014 24747215

[B3] Botvinick M. M. Cohen J. D. Carter C. S. (2004). Conflict monitoring and anterior cingulate cortex: an update. *Trends Cogn. Sci.* 8 539–546. 10.1016/j.tics.2004.10.003 15556023

[B4] Bradley B. P. Mogg K. Wright T. Field M. (2003). Attentional bias in drug dependence: vigilance for cigarette-related cues in smokers. *Psychol. Addict. Behav.* 17 66–72. 10.1037/0893-164X.17.1.66 12665083

[B5] Campbell K. L. Grigg O. Saverino C. Churchill N. Grady C. L. (2013). Age differences in the intrinsic functional connectivity of default network subsystems. *Front. Aging Neurosci.* 5:73. 10.3389/fnagi.2013.00073 24294203 PMC3827623

[B6] Carretié L. Hinojosa J. A. Martín-Loeches M. Mercado F. Tapia M. (2004). Automatic attention to emotional stimuli: neural correlates. *Hum. Brain Mapp.* 22 290–299. 10.1002/hbm.20037 15202107 PMC6871850

[B7] Chanon V. W. Sours C. R. Boettiger C. A. (2010). Attentional bias toward cigarette cues in active smokers. *Psychopharmacology* 212 309–320. 10.1007/s00213-010-1953-1 20668841 PMC2967198

[B8] Clerkin E. M. Magee J. C. Wells T. T. Beard C. Barnett N. P. (2016). Randomized controlled trial of attention bias modification in a racially diverse, socially anxious, alcohol dependent sample. *Behav. Res. Ther.* 87 58–69. 10.1016/j.brat.2016.08.010 27591918 PMC5127758

[B9] Constantinou N. Morgan C. J. Battistella S. O’Ryan D. Davis P. Curran H. V. (2010). Attentional bias, inhibitory control and acute stress in current and former opiate addicts. *Drug Alcohol Depend.* 109 220–225. 10.1016/j.drugalcdep.2010.01.012 20172662

[B10] Corbetta M. Kincade J. M. Ollinger J. M. Mcavoy M. P. Shulman G. L. (2000). Voluntary orienting is dissociated from target detection in human posterior parietal cortex. *Nat. Neurosci.* 3 292–297. 10.1038/73009 10700263

[B11] Cornette L. Dupont P. Salmon E. Orban G. A. (2006). The neural substrate of orientation working memory. *J. Cogn. Neurosci.* 13 813–828. 10.1162/08989290152541476 11564325

[B12] Coullaut-Valera García J. Arbaiza Díaz del Rio I. Coullaut-Valera García R. Ortiz T. (2007). Alterations of P300 wave in occipital lobe in depressive patients. *Actas Esp. Psiquiatr.* 35 243–248. 17592786

[B13] Crunelle C. L. Veltman D. J. Booij J. Brink W. V. D. (2012). Substrates of neuropsychological functioning in stimulant dependence: a review of functional neuroimaging research. *Brain Behav.* 2 499–523. 22950052 10.1002/brb3.65 PMC3432971

[B14] Delorme A. Makeig S. (2004). EEGLAB: an open source toolbox for analysis of single-trial EEG dynamics including independent component analysis. *J. Neurosci. Methods* 134 9–21. 10.1016/j.jneumeth.2003.10.009 15102499

[B15] Donchin E. (1979). “Event-related brain potentials: a tool in the study of human information processing,” in *Evoked Potentials and Behavior* ed. Begleiter H. (New York, NY: Plenum Press) 13–75.

[B16] Donchin E. (1981). Surprise!… Surprise? *Psychophysiology* 18 493–513. 10.1111/j.1469-8986.1981.tb01815.x 7280146

[B17] Dunn B. R. Dunn D. A. Languis M. Andrews D. (1998). The relation of ERP components to complex memory processing. *Brain Cogn.* 36 355–376. 10.1006/brcg.1998.0998 9647684

[B18] Ehrman R. N. Robbins S. J. Bromwell M. A. Lankford M. E. Monterosso J. R. O’Brien C. P. (2002). Comparing attentional bias to smoking cues in current smokers, former smokers, and non-smokers using a dot-probe task. *Drug Alcohol Depend.* 67 185–191. 10.1016/S0376-8716(02)00065-0 12095668

[B19] Fashler S. R. Joel K. (2016). Keeping an eye on pain: investigating visual attention biases in individuals with chronic pain using eye-tracking methodology. *J. Pain Res.* 9 551–561. 27570461 10.2147/JPR.S104268 PMC4986909

[B20] Field M. Cox W. M. (2008). Attentional bias in addictive behaviors: a review of its development, causes, and consequences. *Drug Alcohol Depend.* 97 1–20. 10.1016/j.drugalcdep.2008.03.030 18479844

[B21] Field M. Munafò M. R. Franken I. H. (2009). A meta-analytic investigation of the relationship between attentional bias and subjective craving in substance abuse. *Psychol. Bull.* 135 589–607. 10.1037/a0015843 19586163 PMC2999821

[B22] Franken I. H. (2003). Drug craving and addiction: integrating psychological and neuropsychopharmacological approaches. *Prog. Neuropsychopharmacol. Biol. Psychiatry* 27 563–579. 12787841 10.1016/S0278-5846(03)00081-2

[B23] Franken I. H. Kroon L. Y. Hendriks V. M. (2000). Influence of individual differences in craving and obsessive cocaine thoughts on attentional processes in cocaine abuse patients. *Addict. Behav.* 25 99–102. 10.1016/S0306-4603(98)00112-9 10708323

[B24] Franken I. H. A. Stam C. J. Hendriks V. M. Brink W. V. D. (2003). Neurophysiological evidence for abnormal cognitive processing of drug cues in heroin dependence. *Psychopharmacology* 170 205–212. 10.1007/s00213-003-1542-7 12898125

[B25] Fu L. P. Bi G. H. Zou Z. T. Wang Y. Ye E. M. Ma L. (2008). Impaired response inhibition function in abstinent heroin dependents: an fMRI study. *Neurosci. Lett.* 438 322–326. 10.1016/j.neulet.2008.04.033 18485592

[B26] Fuchs M. Kastner J. Wagner M. Hawes S. Ebersole J. S. (2002). A standardized boundary element method volume conductor model. *Clin. Neurophysiol.* 113 702–712. 10.1016/S1388-2457(02)00030-5 11976050

[B27] Gajewski P. D. Falkenstein M. (2015). Lifelong physical activity and executive functions in older age assessed by memory based task switching. *Neuropsychologia* 73 195–207. 10.1016/j.neuropsychologia.2015.04.031 25937323

[B28] Garcíablanco A. Salmerón L. Perea M. Livianos L. (2014). Attentional biases toward emotional images in the different episodes of bipolar disorder: an eye-tracking study. *Psychiatry Res.* 215 628–633. 10.1016/j.psychres.2013.12.039 24439518

[B29] Gardini S. (2009). Decreased drug-cue-induced attentional bias in individuals with treated and untreated drug dependence. *Acta Neuropsychiatr.* 21 179–185. 10.1111/j.1601-5215.2009.00389.x 25384631

[B30] Gillebert C. R. Dyrholm M. Vangkilde S. Kyllingsbæk S. Peeters R. Vandenberghe R. (2012). Attentional priorities and access to short-term memory: parietal interactions. *Neuroimage* 62 1551–1562. 10.1016/j.neuroimage.2012.05.038 22634216

[B31] Goldstein R. Z. Volkow N. D. (2011). Dysfunction of the prefrontal cortex in addiction: neuroimaging findings and clinical implications. *Nat. Rev. Neurosci.* 12 652–669. 10.1038/nrn3119 22011681 PMC3462342

[B32] Herrmann C. S. Knight R. T. (2001). Mechanisms of human attention: event-related potentials and oscillations. *Neurosci. Biobehav. Rev.* 25 465–476. 10.1016/S0149-7634(01)00027-6 11595268

[B33] Howard R. C. Chaiwutikornwanich A. (2006). The relationship of interrogative suggestibility to memory and attention: an electrophysiological study. *J. Psychophysiol.* 20 79–93. 10.1027/0269-8803.20.2.79

[B34] Hu X. Pornpattananangkul N. Rosenfeld J. P. (2013). N200 and P300 as orthogonal and integrable indicators of distinct awareness and recognition processes in memory detection. *Psychophysiology* 50 454–464. 10.1111/psyp.12018 23317115

[B35] Ibanez A. Melloni M. Huepe D. Helgiu E. Rivera-Rei A. Canales-Johnson A. (2012). What event-related potentials (ERPs) bring to social neuroscience? *Soc. Neurosci.* 7 632–649. 10.1080/17470919.2012.691078 22642412

[B36] Janes A. C. Pizzagalli D. A. Richardt S. Frederick Bde B. Holmes A. J. Sousa J. (2010). Neural substrates of attentional bias for smoking-related cues: an FMRI study. *Neuropsychopharmacology* 35 2339–2345. 10.1038/npp.2010.103 20703221 PMC2955848

[B37] Jatoi M. A. Kamel N. Malik A. S. Faye I. (2014). EEG based brain source localization comparison of sLORETA and eLORETA. *Australas. Phys. Eng. Sci. Med.* 37 713–721. 10.1007/s13246-014-0308-3 25359588

[B38] Kanske P. Plitschka J. Kotz S. A. (2011). Attentional orienting towards emotion: P2 and N400 ERP effects. *Neuropsychologia* 49 3121–3129. 10.1016/j.neuropsychologia.2011.07.022 21816167

[B39] Kappenman E. S. Farrens J. L. Luck S. J. Proudfit G. H. (2014). Behavioral and ERP measures of attentional bias to threat in the dot-probe task: poor reliability and lack of correlation with anxiety. *Front. Psychol.* 5:1368. 10.3389/fpsyg.2014.01368 25538644 PMC4255626

[B40] Kilts C. D. Schweitzer J. B. Quinn C. K. Gross R. E. Faber T. L. Muhammad F. (2001). Neural activity related to drug craving in cocaine addiction. *Arch. Gen. Psychiatry* 58 334–341. 10.1001/archpsyc.58.4.334 11296093

[B41] Klein A. A. Nelson L. M. Anker J. J. (2013). Attention and recognition memory bias for alcohol-related stimuli among alcohol-dependent patients attending residential treatment. *Addict. Behav.* 38 1687–1690. 10.1016/j.addbeh.2012.10.006 23254219

[B42] Kompatsiari K. Candrian G. Mueller A. (2016). Test-retest reliability of ERP components: a short-term replication of a visual Go/NoGo task in ADHD subjects. *Neurosci. Lett.* 617 166–172. 10.1016/j.neulet.2016.02.012 26861197

[B43] Kuo B. C. Stokes M. G. Murray A. M. Nobre A. C. (2014). Attention biases visual activity in visual short-term memory. *J. Cogn. Neurosci.* 26 1377–1389. 10.1162/jocn_a_00577 24456394

[B44] Lauritzen T. Z. D’Esposito M. Heeger D. J. Silver M. A. (2009). Top-down flow of visual spatial attention signals from parietal to occipital cortex. *J. Vis.* 9:18. 10.1167/9.13.18 20055551 PMC2857595

[B45] Lee T. M. Zhou W. H. Luo X. J. Yuen K. S. Ruan X. Z. Weng X. C. (2005). Neural activity associated with cognitive regulation in heroin users: a fMRI study. *Neurosci. Lett.* 382 211–216. 10.1016/j.neulet.2005.03.053 15925092

[B46] Lefebvre C. D. Marchand Y. Eskes G. A. Connolly J. F. (2005). Assessment of working memory abilities using an event-related brain potential (ERP)-compatible digit span backward task. *Clin. Neurophysiol.* 116 1665–1680. 10.1016/j.clinph.2005.03.015 15908268

[B47] Li X. Lu Y. Zhao H. (2014). How and when predictability interacts with accentuation in temporally selective attention during speech comprehension. *Neuropsychologia* 64 (Suppl. 3), 71–84. 10.1016/j.neuropsychologia.2014.09.020 25250708

[B48] Lithfous S. Dufour A. Blanc F. Després O. (2014). Allocentric but not egocentric orientation is impaired during normal aging: an ERP study. *Neuropsychology* 28 761–771. 10.1037/neu0000084 24749730

[B49] Liu Q. Farahibozorg S. Porcaro C. Wenderoth N. Mantini D. (2017). Detecting large-scale networks in the human brain using high-density electroencephalography. *Hum. Brain Mapp.* 38 4631–4643. 10.1002/hbm.23688 28631281 PMC6867042

[B50] Lobben M. D’Ascenzo S. (2015). Grounding grammatical categories: attention bias in hand space influences grammatical congruency judgment of Chinese nominal classifiers. *Front. Psychol.* 6:1299. 10.3389/fpsyg.2015.01299 26379611 PMC4550751

[B51] Lubman D. I. Allen N. B. Peters L. A. Deakin J. F. (2008). Electrophysiological evidence that drug cues have greater salience than other affective stimuli in opiate addiction. *J. Psychopharmacol.* 22 836–842. 10.1177/0269881107083846 18208907

[B52] Lubman D. I. Peters L. A. Mogg K. Bradley B. P. Deakin J. F. (2000). Attentional bias for drug cues in opiate dependence. *Psychol. Med.* 30 169–175. 10.1017/S0033291799001269 10722187

[B53] Luck S. J. Heinze H. J. Mangun G. R. Hillyard S. A. (1990). Visual event-related potentials index focused attention within bilateral stimulus arrays. II. Functional dissociation of P1 and N1 components. *Electroencephalogr. Clin. Neurophysiol.* 75 528–542. 10.1016/0013-4694(90)90139-B 1693897

[B54] Luedke A. Fernandez-Ruiz J. Tam A. Garcia A. (2013). Fractional anisotropy differences in attention and default-mode network areas between healthy aging and Alzheimer’s disease. *Alzheimers Dement.* 9 P601–P602. 10.1016/j.jalz.2013.05.1201

[B55] MacLeod C. Mathews A. Tata P. (1986). Attentional bias in emotional disorders. *J. Abnorm. Psychol.* 95 15–20. 10.1037/0021-843X.95.1.15 3700842

[B56] Marissen M. A. Franken I. H. Waters A. J. Blanken P. van den Brink W. Hendriks V. M. (2006). Attentional bias predicts heroin relapse following treatment. *Addiction* 101 1306–1312. 10.1111/j.1360-0443.2006.01498.x 16911730

[B57] Mayer A. R. Wilcox C. E. Dodd A. B. Klimaj S. D. Dekonenko C. J. Claus E. D. (2016). The efficacy of attention bias modification therapy in cocaine use disorders. *Am. J. Drug Alcohol Abuse* 42 459–468. 10.3109/00952990.2016.1151523 27184297 PMC4979538

[B58] Mcateer A. M. Curran D. Hanna D. (2015). Alcohol attention bias in adolescent social drinkers: an eye tracking study. *Psychopharmacology* 232 3183–3191. 10.1007/s00213-015-3969-z 26014111

[B59] Mckay J. R. (1999). Studies of factors in relapse to alcohol, drug and nicotine use: a critical review of methodologies and findings. *J. Stud. Alcohol* 60 566–576. 10.15288/jsa.1999.60.566 10463814

[B60] Michalewski H. J. Prasher D. K. Starr A. (1986). Latency variability and temporal interrelationships of the auditory event-related potentials (N1 P2 N2 and P3) in normal subjects. *Electroencephalogr. Clin. Neurophysiol.* 65 59–71. 2416547 10.1016/0168-5597(86)90037-7

[B61] Molenberghs P. Gapp J. Wang B. Louis W. R. Decety J. (2016). Increased moral sensitivity for outgroup perpetrators harming ingroup members. *Cereb. Cortex* 26 225–233. 25183886 10.1093/cercor/bhu195

[B62] Molenberghs P. Mesulam M. M. Peeters R. Vandenberghe R. R. (2007). Remapping attentional priorities: differential contribution of superior parietal lobule and intraparietal sulcus. *Cereb. Cortex* 17 2703–2712. 10.1093/cercor/bhl179 17264251

[B63] Nieuwenhuis S. Yeung N. Wery V. D. W. Ridderinkhof K. R. (2003). Electrophysiological correlates of anterior cingulate function in a go/no-go task: effects of response conflict and trial type frequency. *Cogn. Affect. Behav. Neurosci.* 3 17–26. 10.3758/CABN.3.1.17 12822595

[B64] Nobre A. C. Griffin I. C. Rao A. (2008). Spatial attention can bias search in visual short-term memory. *Front. Hum. Neurosci.* 1:4. 10.3389/neuro.09.004.2007 18958218 PMC2525979

[B65] Noël X. Colmant M. Van D. L. M. Bechara A. Bullens Q. Hanak C. (2006). Time course of attention for alcohol cues in abstinent alcoholic patients: the role of initial orienting. *Alcohol. Clin. Exp. Res.* 30 1871–1877. 10.1111/j.1530-0277.2006.00224.x 17067351

[B66] Norman L. Lawrence N. Iles A. Benattayallah A. Karl A. (2014). Attachment-security priming attenuates amygdala activation to social and linguistic threat. *Soc. Cogn. Affect. Neurosci.* 10 832–839. 25326039 10.1093/scan/nsu127 PMC4448028

[B67] Omoto S. Kuroiwa Y. Otsuka S. Baba Y. Wang C. Li M. (2010). P1 and P2 components of human visual evoked potentials are modulated by depth perception of 3-dimensional images. *Clin. Neurophysiol.* 121 386–391. 10.1016/j.clinph.2009.12.005 20071231

[B68] Oren N. Shapiralichter I. Lerner Y. Tarrasch R. Hendler T. Giladi N. (2016). How attention modulates encoding of dynamic stimuli. *Front. Hum. Neurosci.* 10:507 10.3389/fnhum.2016.00507 27818628 PMC5073125

[B69] Pandria N. Kovatsi L. Vivas A. B. Bamidis P. D. (2016). Resting-state abnormalities in heroin-dependent individuals. *Neuroscience* [Epub ahead of print]. 10.1016/j.neuroscience.2016.11.018 27884551

[B70] Pascual-Marqui R. D. (2002). Standardized low-resolution brain electromagnetic tomography (sLORETA): technical details. *Methods Find. Exp. Clin. Pharmacol.* 24 (Suppl. D), 5–12. 12575463

[B71] Pascualmarqui R. D. Lehmann D. Koukkou M. Kochi K. Anderer P. Saletu B. (2011). Assessing interactions in the brain with exact low-resolution electromagnetic tomography. *Philos. Trans. R. Soc. A Math. Phys. Eng. Sci.* 369 3768–3784. 10.1098/rsta.2011.0081 21893527

[B72] Pflugshaupt T. Nösberger M. Gutbrod K. Weber K. P. Linnebank M. Brugger P. (2016). Bottom-up visual integration in the medial parietal lobe. *Cereb. Cortex* 26 943–949. 10.1093/cercor/bhu256 25331599

[B73] Pinal D. Zurrón M. Díaz F. (2014). Effects of load and maintenance duration on the time course of information encoding and retrieval in working memory: from perceptual analysis to post-categorization processes. *Front. Hum. Neurosci.* 8:165. 10.3389/fnhum.2014.00165 24744715 PMC3978287

[B74] Polich J. (2007). Updating P300: an integrative theory of P3a and P3b. *Clin. Neurophysiol.* 118 2128–2148. 10.1016/j.clinph.2007.04.019 17573239 PMC2715154

[B75] Rahmanian M. Mirjafari S. A. Hasani J. (2006). The relationship between craving and attentional bias in opioid dependent, relapsed and abstinent individuals. *Iran. J. Psychiatry Clin. Psychol.* 12 216–22.

[B76] Richards J. E. (2003). Attention affects the recognition of briefly presented visual stimuli in infants: an ERP study. *Dev. Sci.* 6 312–328. 10.1111/1467-7687.00287 16718304 PMC1464402

[B77] Robinson T. E. Berridge K. C. (2008). Review. The incentive sensitization theory of addiction: some current issues. *Philos. Trans. R. Soc. Lond. B Biol. Sci.* 363 3137–3146. 10.1098/rstb.2008.0093 18640920 PMC2607325

[B78] Rosazza C. Cai Q. Minati L. Paulignan Y. Nazir T. A. (2009). Early involvement of dorsal and ventral pathways in visual word recognition: an ERP study. *Brain Res.* 1272 32–44. 10.1016/j.brainres.2009.03.033 19332032

[B79] Schmidt A. Borgwardt S. Gerber H. Schmid O. Wiesbeck G. A. Riecherrössler A. (2014). Altered prefrontal connectivity after acute heroin administration during cognitive control. *Int. J. Neuropsychopharmacol.* 17 1375–1385. 10.1017/S1461145714000297 24641978

[B80] Serences J. T. Shomstein S. Leber A. B. Golay X. Egeth H. E. Yantis S. (2005). Coordination of voluntary and stimulus-driven attentional control in human cortex. *Psychol. Sci.* 16 114–122. 10.1111/j.0956-7976.2005.00791.x 15686577

[B81] Shechner T. Jarcho J. M. Britton J. C. Leibenluft E. Pine D. S. Nelson E. E. (2013). Attention bias of anxious youth during extended exposure of emotional face pairs: an eye-tracking study. *Depress. Anxiety* 30 14–21. 10.1002/da.21986 22815254 PMC3541440

[B82] Spencer S. (2015). *Investigation of Attention Bias to Cigarette-Related Cues Among Cigarette Smokers.* Milwaukee, WI: University of Wisconsin–Milwaukee.

[B83] Thai N. Taber-Thomas B. C. Pérez-Edgar K. E. (2016). Neural correlates of attention biases, behavioral inhibition, and social anxiety in children: an ERP study. *Dev. Cogn. Neurosci.* 19 200–210. 10.1016/j.dcn.2016.03.008 27061248 PMC4912890

[B84] Townshend J. M. Duka T. (2001). Attentional bias associated with alcohol cues: differences between heavy and occasional social drinkers. *Psychopharmacology* 157 67–74. 10.1007/s002130100764 11512045

[B85] Tucker A. M. Rakitin B. C. Basner R. C. Gazes Y. Steffener J. Stern Y. (2011). fMRI activation during failures to respond key to understanding performance changes with sleep deprivation. *Behav. Brain Res.* 218 73–79. 10.1016/j.bbr.2010.11.012 21074577 PMC3022081

[B86] Ursache A. Blair C. (2015). Children’s cortisol and salivary alpha-amylase interact to predict attention bias to threatening stimuli. *Physiol. Behav.* 138 266–272. 10.1016/j.physbeh.2014.10.002 25455863 PMC5241704

[B87] Vandenberghe R. Molenberghs P. Gillebert C. R. (2012). Spatial attention deficits in humans: the critical role of superior compared to inferior parietal lesions. *Neuropsychologia* 50 1092–1103. 10.1016/j.neuropsychologia.2011.12.016 22266260

[B88] Verleger R. (1988). Event-related potentials and cognition: a critique of the context updating hypothesis and an alternative interpretation of P3. *Behav. Brain Sci.* 11 343–356. 10.1017/S0140525X00058015

[B89] Wang Y. Ma H. Fu S. Guo S. Yang X. Luo P. (2014). Long-term exposure to high altitude affects voluntary spatial attention at early and late processing stages. *Sci. Rep.* 4:4443 10.1038/srep04443

[B90] Wang Y. Xu P. Jiang Y. (2007). An EPR study on attention bias for addictive and emotional cues in abstinent heroin addicts. *Psychol. Sci.* 30 202–191.

[B91] Warbrick T. Arrubla J. Boers F. Neuner I. Shah N. J. (2014). Attention to detail: why considering task demands is essential for single-trial analysis of BOLD correlates of the visual P1 and N1. *J. Cogn. Neurosci.* 26 529–542. 10.1162/jocn_a_00490 24047390

[B92] Waters A. J. Marhe R. Franken I. H. (2012). Attentional bias to drug cues is elevated before and during temptations to use heroin and cocaine. *Psychopharmacology* 219 909–921. 10.1007/s00213-011-2424-z 21833505 PMC4350583

[B93] Wilkinson D. T. Halligan P. W. Henson R. N. Dolan R. J. (2002). The effects of interdistracter similarity on search processes in superior parietal cortex. *Neuroimage* 15 611–619. 10.1006/nimg.2001.0993 11848704

[B94] Wu H. Hu X. Fu G. (2009). Does willingness affect the N2-P3 effect of deceptive and honest responses? *Neurosci. Lett.* 467 63–66. 10.1016/j.neulet.2009.10.002 19818837

[B95] Zhao Q. Hua J. Hu B. Li Y. Ning Z. Mi L. (2017). Nonlinear dynamic complexity and sources of resting-state eeg in abstinent heroin addicts. *IEEE Trans. Nanobiosci.* 16 349–355. 28809667 10.1109/TNB.2017.2705689

