# Supplementary material for: Neural Correlates of Drug-Related Attentional Bias in Heroin Dependence
Source: Front Hum Neurosci. 2018 Jan 23;11:646. doi: 10.3389/fnhum.2017.00646 (PMC5787086; doi:10.3389/fnhum.2017.00646)
Supplement: Supplementary file 1 [file Data_Sheet_1.docx]

Supplementary Material

Article Title Neural correlates of drug-related attentional bias in heroin dependence

Qinglin Zhao, Bin Hu, Quanying Liu

*** Correspondence:** [bh@lzu.edu.cn](mailto:bh@lzu.edu.cn) ; [quanying.liu@hest.ethz.ch](mailto:quanying.liu@hest.ethz.ch)

# Supplementary Figures and Tables

## Supplementary Figures


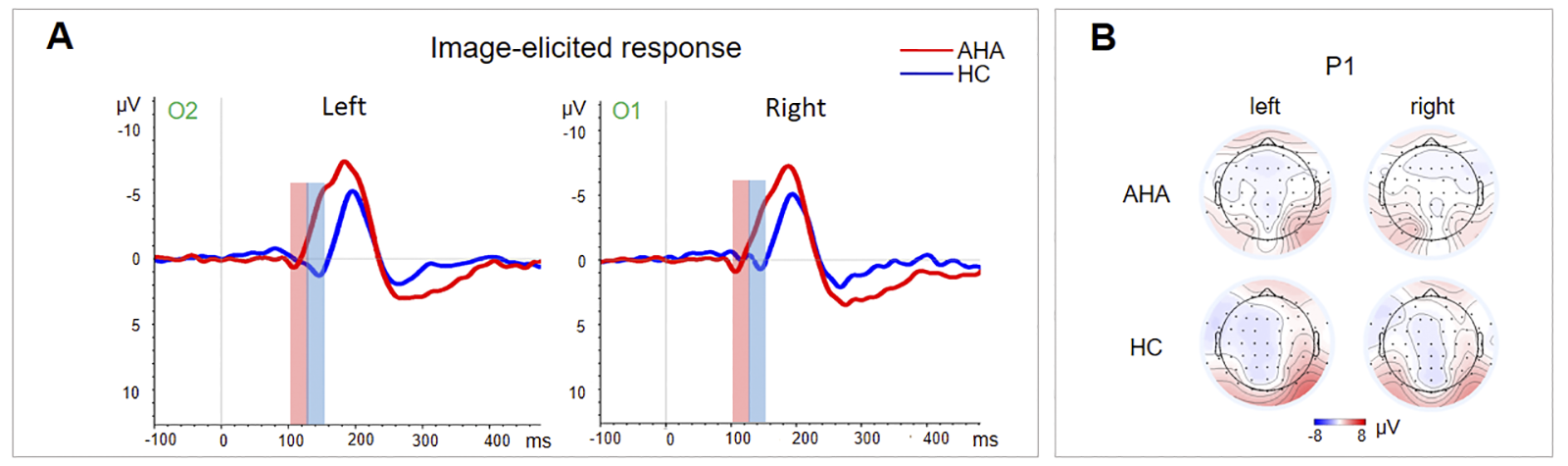


**Figure S1 – Image-elicited P1 component.** (A) ERP waveform per condition and group. The P1 time window for AHA and HC group is marked in red and blue shadow, respectively. (B) Scalp topography of the P1 component for the drug-related cue presented on the left or right screen for AHA and HC, respectively.


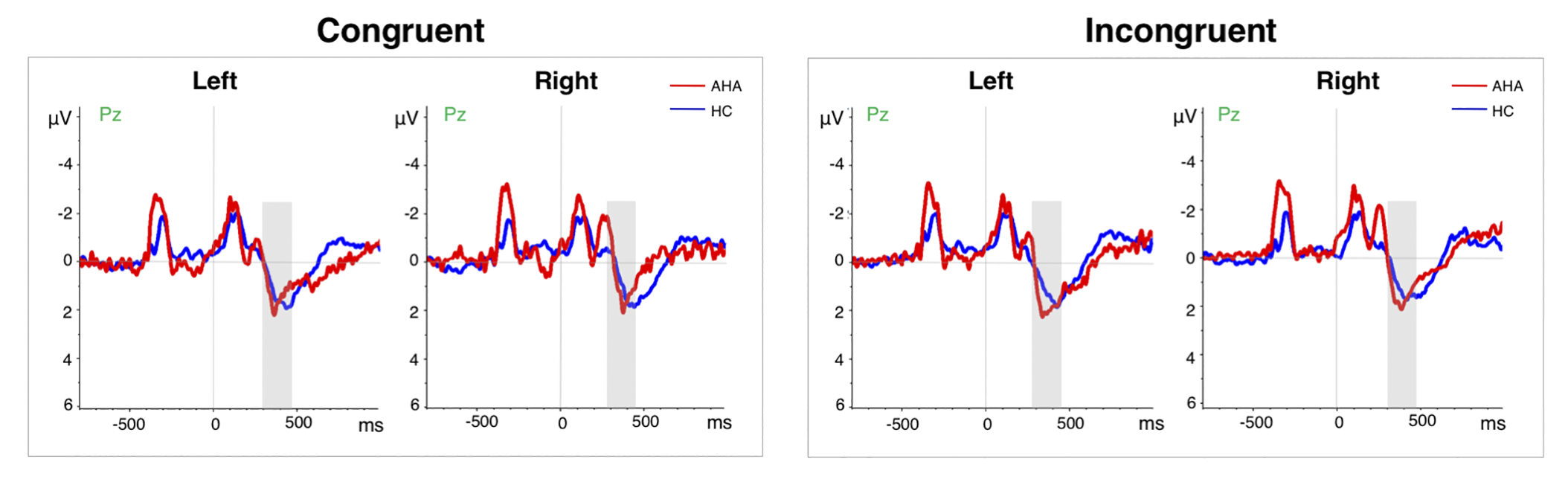


**Figure S2 – Target-induced P3 components.** The full trial ERP waveforms per condition and per group during were illustrated. Importantly, to avoid the carry-over effect of image-induced response, the target-locked data were baseline corrected using the average voltage of 700ms to 500ms before dot stimulus onset (200ms to 0ms before image stimulus onset). The time window for P3 component for each case is marked by the gray shadow.

| **Table S1 - Peak MNI coordinates for within-subject factor comparisons. Clusters surviving threshold of p<0.05 FWE-corrected.** | | | | | | | | | | | | |
| --- | --- | --- | --- | --- | --- | --- | --- | --- | --- | --- | --- | --- |
| **Component** | **Time (ms)** | **Group** | **Contrast** | **Coordinate Region** | **Peak MNI Coordinate** | | | | | | **P-Value** | |
| P1 | 90-150 | AHA | LEFT vs. RIGHT | PFL | -20 | 5 | | 55 | | | 0.0456^*^ | |
|  | 90-160 | HC | LEFT vs. RIGHT | ACC | 15 | -20 | | 45 | | | 0.0426^*^ | |
| N1 | 170-220 | AHA | LEFT vs. RIGHT |  |  |  | |  | | | 0.3622 | |
|  | 185-220 | HC | LEFT vs. RIGHT |  |  |  | |  | | | 0.8488 | |
| P2\|N2 | 230-350 | AHA | LEFT vs. RIGHT | Insula | -45 | -40 | | 20 | | | 0.0170^*^ | |
|  | 240-350 | HC | LEFT vs. RIGHT |  |  |  | |  | | | 0.6888 | |
| P3 | 370-450 | AHA | CON vs. INCON | dlPFL  SFG  dACC  IPL | -25  -20  -20  -55 | 15  10  5  -40 | | 50  55  50  30 | | | 0.0088^*^ | |
|  | 390-490 | HC | CON vs. INCON |  | | |  | |  |  | | 0.82440 |
| *Abbreviations: ACC, anterior cingulate cortex; dACC: dorsal anterior cingulate cortex; PFL: posterior frontal lobe; SFG: superior frontal gyrus; dlPFL: dorsolateral prefrontal lobe; IPL: inferior parietal lobe; CON: congruent; INCON: incongruent* | | | | | | | | | | | | |
