# Supplementary figures and images for: Neural Correlates of Drug-Related Attentional Bias in Heroin Dependence
Source: Front Hum Neurosci. 2018 Jan 23;11:646. doi: 10.3389/fnhum.2017.00646 (PMC5787086; doi:10.3389/fnhum.2017.00646)

# A

## Image-elicited response

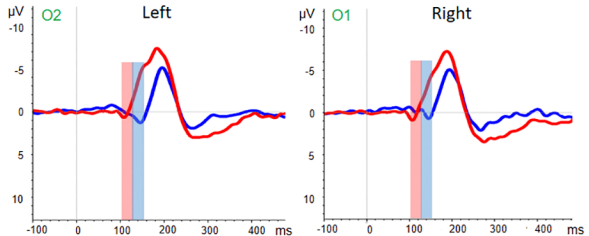

# B

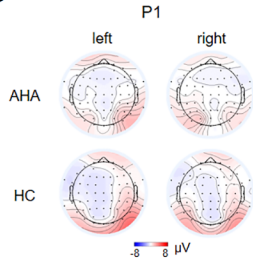

Supplement: Supplementary file 2 [file Image_1.PDF]

## Congruent

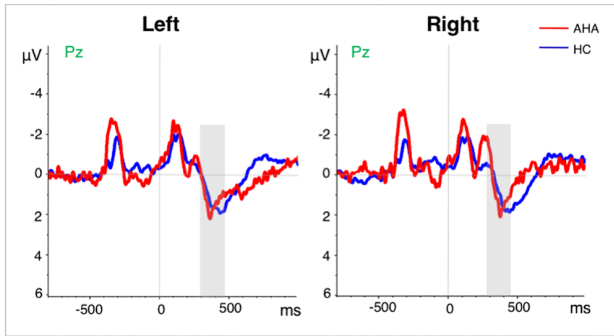

## Incongruent

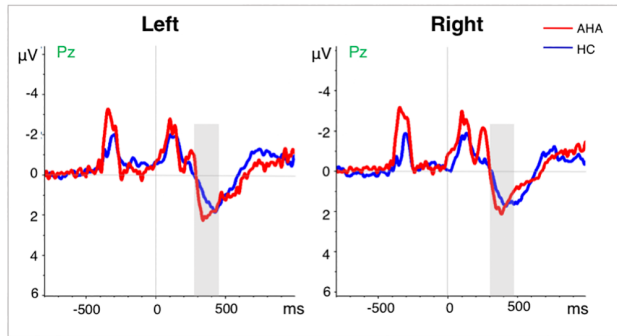

Supplement: Supplementary file 3 [file Image_2.PDF]
